# Automated Radiographic Measurements of Knee Osteoarthritis

**DOI:** 10.1177/19476035231166126

**Published:** 2023-06-02

**Authors:** H. Rayegan, H.C. Nguyen, H. Weinans, W.P. Gielis, S.Y. Ahmadi Brooghani, R.J.H. Custers, N. van Egmond, C. Lindner, V. Arbabi

**Affiliations:** 1 Orthopaedic-BioMechanics Research Group, University of Birjand, Birjand, Iran; 2 Department of Mechanical Engineering, Faculty of Engineering, University of Birjand, Birjand, Iran; 3 Department of Orthopaedic Surgery, University Medical Center Utrecht, Utrecht, The Netherlands; 4 3D Lab, University Medical Centre Utrecht, Utrecht, The Netherlands; 5 Department of Biomechanical Engineering, Faculty of Mechanical, Maritime and Materials Engineering (3mE), Delft University of Technology, Delft, The Netherlands; 6 Division of Informatics, Imaging & Data Sciences, The University of Manchester, Manchester, UK

**Keywords:** measurements, automation, osteoarthritis, knee, radiological imaging

## Abstract

**Objective:**

Herewith, we report the development of Orthopedic Digital Image Analysis (ODIA) software that is developed to obtain quantitative measurements of knee osteoarthritis (OA) radiographs automatically. Manual segmentation and measurement of OA parameters currently hamper large-cohort analyses, and therefore, automated and reproducible methods are a valuable addition in OA research. This study aims to test the automated ODIA measurements and compare them with available manual Knee Imaging Digital Analysis (KIDA) measurements as comparison.

**Design:**

This study included data from the CHECK (Cohort Hip and Cohort Knee) initiative, a prospective multicentre cohort study in the Netherlands with 1,002 participants. Knee radiographs obtained at baseline of the CHECK cohort were included and mean medial/lateral joint space width (JSW), minimal JSW, joint line convergence angle (JLCA), eminence heights, and subchondral bone intensities were compared between ODIA and KIDA.

**Results:**

Of the potential 2,004 radiographs, 1,743 were included for analyses. Poor intraclass correlation coefficients (ICCs) were reported for the JLCA (0.422) and minimal JSW (0.299). The mean medial and lateral JSW, eminence height, and subchondral bone intensities reported a moderate to good ICC (0.7 or higher). Discrepancies in JLCA and minimal JSW between the 2 methods were mostly a problem in the lateral tibia plateau.

**Conclusions:**

The current ODIA tool provides important measurements of OA parameters in an automated manner from standard radiographs of the knee. Given the automated and computerized methodology that has very high reproducibility, ODIA is suitable for large epidemiological cohorts with various follow-up time points to investigate structural progression, such as CHECK or the Osteoarthritis Initiative (OAI).

## Introduction

Osteoarthritis (OA) is estimated to affect worldwide 10% of men and 18% of women aged over 60 years^1^ with an enormous burden for patients and societal health care costs.^1^ Of the total OA burden worldwide, approximately 85% is accounted for by knee OA.^2^ OA is a whole joint disease, with structural changes in the hyaline articular cartilage, subchondral bone, ligaments, capsule, synovium, and periarticular muscles.^2^ The complex pathogenesis of OA ultimately leads to structural destruction and failure of the joint. The disease is an active dynamic alteration caused by an imbalance between repair and destruction of joint tissues, due to inflammatory, mechanical, and metabolic factors.^2^

Gold standard for diagnosing knee OA are standard knee radiographs, which are useful for monitoring disease progression due to the possibility of frequent imaging.^3^ However, knee radiographs combined with current grading systems are not sensitive enough to detect early signs of disease progression.^3^-^5^ Joint space width (JSW) measurements on knee radiographs are an important parameter when monitoring OA progression, especially minimal JSW.^3,6^ Femoro-tibial angles (FTAs) from standard knee radiographs are strongly correlated with hip-knee-ankle angles (HKAs), and are together with the joint line convergence angle (JLCA) an important indicator for malalignment-induced OA.^1,7,8^ Early stage OA is associated with changes in subchondral bone mineralization, bone volume, increased bone turnover, and vascular invasion.^1,2,9^ These alterations possibly affect subchondral bone intensities and eminence heights on radiographs and could therefore improve diagnostics.^2,9^

To improve the sensitivity of monitoring disease progression in OA research, quantification of radiographical knee OA measurements in continuous variables has been introduced by proposing the use of Knee Imaging Digital Analysis (KIDA) software to better quantify knee OA status and progression.^3^ However, KIDA is relatively time-intensive and somewhat reader-dependent due to its manual segmentation procedure of different bone shapes and knee OA measurements,^3^ and analyses of large clinical cohorts such as the Osteoarthritis Initiative (OAI) are therefore hampered.^10^ Quick and accurate measurements on radiographs are important future radiographical studies.^2,4,11^ Ideally, a computer program automatically annotates the bones on knee radiographs with important landmarks, whereafter automated measurements of interest can be performed quickly and accurately.

Herewith we report the development of Orthopedic Digital Image Analysis (ODIA) software that is developed to obtain quantitative measurements of knee OA radiographs in a fast, automated, and consistent manner. The start of the ODIA software is a number of landmark points automatically placed on standard knee radiographs by BoneFinder® (www.bone-finder.com, The University of Manchester, UK).^12,13^ The automatically annotated points are subsequently used to identify specific locations and perform automated measurements of OA parameters.

This study aimed to develop an automated workflow to measure knee OA parameters. The hypothesis is that such an automated method can perform measurements reproducibly and quickly in large epidemiological study cohorts such as CHECK^14^ or OAI.^15^

## Materials and Methods

### Selections and Description of the Population

This study included data from the CHECK (Cohort Hip and Cohort Knee) initiative, a prospective nationwide multicentre longitudinal cohort study (10 general and academic hospitals) in the Netherlands with 1,002 participants.^14^ Medical ethics committees of all participating centers approved the study, and all participants gave their written informed consent.^14^ Patients presenting pain and/or stiffness in their hip(s) and/or knee(s) at their general practitioner were referred to 1 of the 10 participating hospitals in the Netherlands.

### Inclusion and Exclusion Criteria

Patients were eligible when aged between 45 and 65 at the time of inclusion and had first onset of pain and/or stiffness in the hip(s) and/or knee(s). Both groups were included in this study. Also, patients had never or no longer than 6 months ago consulted a physician for these complaints. When presenting a pathological condition other than OA that could explain the symptoms (e.g., other rheumatic disease, previous hip or knee joint replacement, congenital dysplasia, osteochondritis dissecans, intra-articular fractures, septic arthritis, Perthes’ disease, ligament or meniscus damage, plica syndrome, Baker’s cyst), the patient was excluded.^15^

### Acquisition of the Radiographs and Parameters

**Figure 1.**
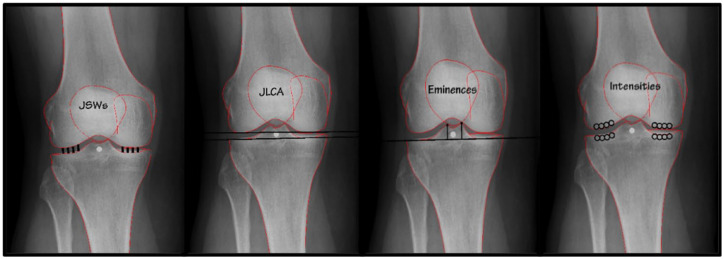
Measured parameters on knee radiographs, from left to right: Joint space width (JSW), joint line convergence angle (JLCA), eminence heights, and subchondral bone intensities.

All knee radiographs were obtained following the Buckland-Wright protocol.^16^ Radiographs were made bilaterally with the knee in a semi-flexed position (7°-10°) and weightbearing posteroanterior (PA) view. The current study only included the radiographs obtained at baseline of the CHECK cohort. Included parameters measured on these radiographs were mean medial/lateral JSW, minimal JSW, JLCA, eminence heights, and subchondral bone intensities (**Fig. 1**).

### KIDA

The CHECK initiative contains an available database (Thematic collection: CHECK—EASY [knaw.nl]) with the measurements on knee radiographs shown in **Figure 1** performed with KIDA.^3^ These data were used herewith to evaluate the automated ODIA outcome measures. User input requirements for KIDA, which take up to 10 minutes per radiograph, are meticulously described by Marijnissen *et al.*^3^

### ODIA and Semi-Automated Segmentation (ODIA Semi-Auto)

**Figure 2.**
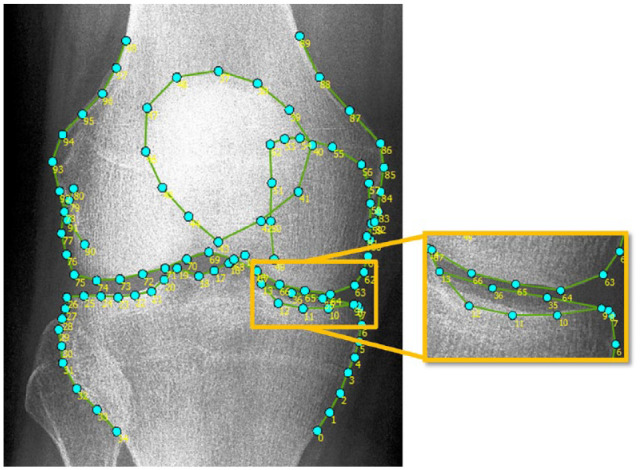
The 99-points model automatically placed on knee radiograph using BoneFinder®.^12,13^ The points on the lateral tibia plateau were placed on the anticipated anterior edge.

ODIA software was developed by our research group using MATLAB (version R2016a, the MathWorks Inc., Natick, MA) and as a first step utilizes landmark points placed by BoneFinder®.^12,13^ Using 99 landmarks, this BoneFinder® model automatically annotates the distal femur, patella, and proximal tibia (**Fig. 2**).^12,13^ A visual check and correction if needed were performed for each radiograph. Manual corrections were performed by an orthopedic researcher (W.P.G.) with 5 years of experience in OA research with a focus on image analysis, in less than 1 minute per radiograph.

**Figure 3.**
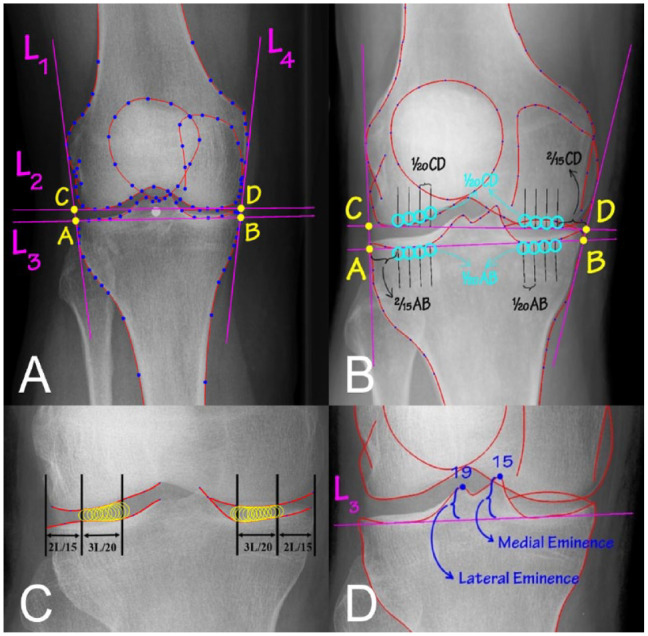
The measurements as performed by the Orthopedic Digital Image Analysis (ODIA). (**A**) The framework placed by ODIA. (**B**) The perpendiculars on the joint lines and the circles for subchondral bone density measurements. The circles were placed directly under the anticipated anterior edge of the tibia plateau. Joint space width (JSW) is measured as the circle diameters displayed in (**C**). The smallest circle diameter is the minimal JSW. Again, the anticipated anterior edge of the tibia plateau was used at the lateral side. Eminence heights are measured as can be seen in (**D**).

After the semi-automatic process of locating and potentially correcting some of the 99 points, the measurements as visualized in **Figure 1** were performed automatically using ODIA. The operating time of ODIA was negligible as the user only must select the OA parameters of interest and run the software. The cohort size does not influence this setup process. To do so, ODIA executed the following steps:

*1. Defining framework on radiograph* (**Fig. 3A**)

ODIA sets 4 lines around the region of interest on the radiograph:

- L1 is a line touching the lateral curves passing from points 24 to 32 for tibia, and 74 to 80 and 90 to 98 for femur.
- L2 is a line touching the femoral condyles passing from points 63 to 68 for medial and 70 to 75 for lateral.
- L3 is a line touching the tibial plateau passing from points 9 to 13 for medial and 20 to 25 for lateral.
- L4 is a line touching the medial curves passing from points 2 to 7 for tibia and 58 to 64 for femur.
- Points A till D are intersections of L2 and L3 with L1 and L4.

*2. Defining knee joint boundaries* (**Fig. 3B**)

- ODIA calculates the position of 2 upward perpendiculars on line L2 in the lateral and medial compartment, with the same procedure downward on line L3.
- The outer perpendiculars are placed on line L3 at 2/15 AB inward from points A and B. The same was done for the outer perpendiculars on line L2, which were placed at 2/15 CD inward from points C and D (**Fig. 3B**).
- The inner perpendiculars are placed at 3/20 from the outer perpendiculars on line L2 and L3^3^ (**Fig. 3B**).
- The bone edges marked by BoneFinder® (anterior lateral and medial tibial plateau, distal femoral condyles) together with the perpendiculars are set as joint boundaries by ODIA.^12,13^

*3. Calculate mean medial/lateral JSW, and minimal JSW* (**Fig. 3C**)

- ODIA fits 30 intra-articular circles between the joint boundaries, with the circle diameters as calculated JSWs.
- The smallest circle diameter at either medial or lateral side is assigned as the minimal JSW.

*4. Calculate mean subchondral bone intensities* (**Fig. 3B**)

- Each radiograph includes a calibration phantom (or wedge) made from aluminum (Al), with known dimensions (mm). ODIA recognizes the phantoms (or wedges) and calibrates the intensity profiles expressed in mmAl.
- Four circles (diameter of 1/20 AB or CD) are placed against the joint boundaries (on the bones) with the centers on 4 perpendiculars. The 4 perpendiculars are placed at a mutual distance 1/20 of AB and CD, respectively (**Fig. 3B**).
- ODIA calculates the mean subchondral bone intensities within those circles defined in mmAl.

*5. JLCA measurement* (**Fig. 3A**)

- Lines L2 and L3 (**Fig. 3A**), formed as the joint boundaries, are identical to the distal femoral and proximal tibial joint lines as defined by Paley.^17^ Therefore, the angle between L2 and L3 calculated by ODIA represents the JLCA.

*6. Eminence height measurements* (**Fig. 3D**)

- Landmark points 15 and 19 are the most proximal points of the tibial eminence. ODIA measured the distance from points 15 and 19 to line L3 (**Fig. 3D**), representing the eminence heights.

**Figure 4.**
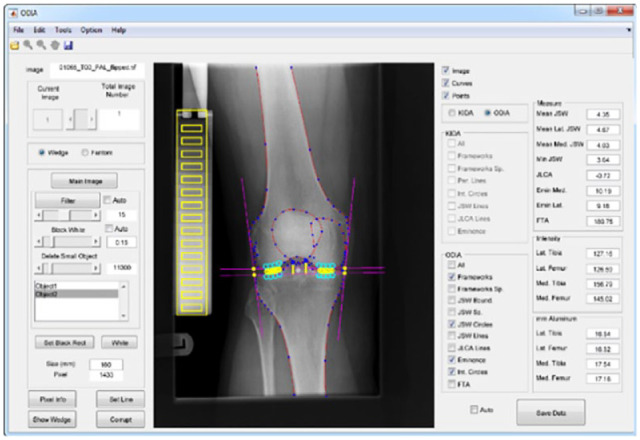
User interface of ODIA software, which performs fully automated measurements of the parameters joint space width (JSW), eminence height, subchondral bone intensity, and joint line convergence angle (JLCA). ODIA = Orthopedic Digital Image Analysis.

The overall workflow is performed within a user interface that visualizes each step and provides outcome parameters upon request (**Fig. 4**).

### ODIA and Full-Automated Segmentation (ODIA Full Auto)

This study also tested a fully automated workflow without manual checks and corrections of the BoneFinder® annotated point positions, meaning the segmentation phase of the radiographs. ODIA full auto has no operating time as the segmentation phase was automated too. Analyzing radiographs using this ODIA full auto method only requires setup time of around 10 minutes, independent of the cohort size.

**Figure 5.**
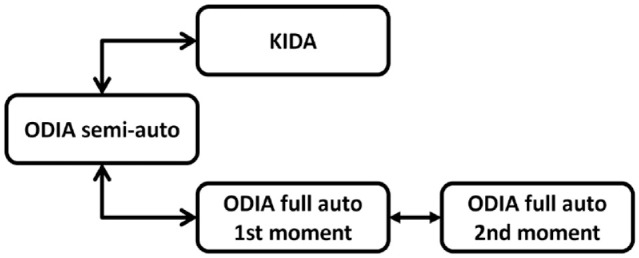
Overview of the 3 measurement methods used in this study. The manual KIDA method, the ODIA semi-auto method with manual checks of the segmentations and automated measurements, and twice the ODIA full auto method with automated segmentations and automated measurements. KIDA = Knee Imaging Digital Analysis; ODIA = Orthopedic Digital Image Analysis.

The BoneFinder® algorithm was not trained on 145 CHECK radiographs on T2. This set was used to test the full-automated method (ODIA full auto) by comparing it with the ODIA semi-auto measurements. The ODIA full auto method was performed twice for reproducibility analyses. **Figure 5** illustrates an overview of the 4 different measurement methods and comparisons between those measurement methods.

### Statistical Analyses

Two-way mixed intraclass correlation coefficient (ICC) tests for absolute agreement were performed for the agreement between KIDA and ODIA. Bland-Altman analyses were used to visualize the mean errors and 95% confidence intervals (CI) between KIDA and ODIA. Errors were reported in absolute means with 95% CI. Statistical significance was set at alpha = 0.05. The reproducibility of ODIA full auto was analyzed using ICC tests for absolute agreements, and the absolute differences with standard deviations (SD) between the 2 measurement moments were calculated. All statistical calculations were performed in SPSS Statistics (IBM, version 25.0.0.2.).

## Results

This study included 1,743 of the potential 2,004 knee radiographs, obtained from 922 patients. A total of 261 radiographs were excluded from analyses where the field of view was too short, making it impossible for segmentations using BoneFinder®.^12,13^ Characteristics of the 1,743 knee radiographs and ODIA measurements are listed in **Table 1**. All knee radiographs were centrally graded in the CHECK cohort according to the Kellgren and Lawrence (KL) classification system, with a meticulously described method in earlier studies.^18,19^

**Table 1.** Characteristics of the Participating Patients.

Characteristic	
Age in years, median (range)	56 (45-65)
Females	67.5%
BMI, mean (SD)	26.5 (4)
KL 0	60.7%
KL 1	28.6%
KL 2	10.6%
KL 3	0.1%
KL 4	0.0%

BMI = body mass index; KL = Kellgren-Lawrence grade.

### KIDA and ODIA Semi-Auto

ICCs between the measured parameters using KIDA and ODIA are listed in **Table 2**. Poor ICCs were reported for the JLCA (0.422) and in particular for minimal JSW (0.299). The mean lateral JSW reported a moderate ICC (0.699). All other parameters reported good ICCs (between 0.759 and 0.810) for the measurements performed on KIDA and ODIA.^20^

**Table 2.** Intraclass Correlations Between the KIDA Manual and ODIA Measurements.

		ODIA (ICC)	95% CI
	Mean lateral JSW	0.699	0.673-0.724
	Mean medial JSW	0.876	0.810-0.914
	Minimal JSW	0.299	–0.066-0.558
	JLCA	0.422	0.350-0.485
KIDA	Lateral eminence height	0.768	0.701-0.817
	Medial eminence height	0.786	0.745-0.820
	Medial tibial subchondral bone intensity	0.811	0.789-0.831
	Lateral tibial subchondral bone intensity	0.759	0.733-0.783
	Medial femoral subchondral bone intensity	0.795	0.773-0.816
	Lateral femoral subchondral bone intensity	0.760	0.734-0.783

KIDA = Knee Imaging Digital Analysis; ODIA = Orthopedic Digital Image Analysis; ICC = 2-way mixed intraclass correlation coefficient; CI = confidence interval; JSW = joint space width; JLCA = joint line convergence angle.

The measured ICCs between KIDA and ODIA outcomes were also analyzed per KL score on baseline. These results can be observed in **Table 3**.

**Table 3.** Intraclass Correlations Between the KIDA Manual and ODIA Measurements, Differentiated Between Kellgren and Lawrence Scores 0, 1, and 2.

		ODIA (ICC)	95% CI
	Mean lateral JSW	0.694	0.660-0.725
	Mean medial JSW	0.859	0.770-0.906
	Minimal JSW	0.256	–0.068-0.505
	JLCA	0.314	0.216-0.401
KIDA	Lateral eminence height	0.764	0.713-0.803
KL 0 (*N* = 1,032)	Medial eminence height	0.771	0.724-0.809
	Medial tibial subchondral bone intensity	0.815	0.788-0.838
	Lateral tibial subchondral bone intensity	0.755	0.720-0.787
	Medial femoral subchondral bone intensity	0.782	0.752-0.809
	Lateral femoral subchondral bone intensity	0.750	0.717-0.781
	Mean lateral JSW	0.716	0.667-0.759
	Mean medial JSW	0.873	0.825-0.905
	Minimal JSW	0.283	–0.061-0.536
	JLCA	0.452	0.364-0.530
KIDA	Lateral eminence height	0.769	0.646-0.841
KL 1 (*n* = 487)	Medial eminence height	0.807	0.756-0.846
	Medial tibial subchondral bone intensity	0.817	0.771-0.854
	Lateral tibial subchondral bone intensity	0.783	0.734-0.723
	Medial femoral subchondral bone intensity	0.792	0.720-0.843
	Lateral femoral subchondral bone intensity	0.759	0.676-0.818
	Mean lateral JSW	0.682	0.552-0.714
	Mean medial JSW	0.909	0.818-0.948
	Minimal JSW	0.394	–0.071-0.674
	JLCA	0.557	0.448-0.650
KIDA	Lateral eminence height	0.764	0.654-0.837
KL 2 (*n* = 180)	Medial eminence height	0.789	0.711-0.846
	Medial tibial subchondral bone intensity	0.850	0.774-0.901
	Lateral tibial subchondral bone intensity	0.803	0.714-0.867
	Medial femoral subchondral bone intensity	0.829	0.708-0.896
	Lateral femoral subchondral bone intensity	0.779	0.602-0.870

KIDA = Knee Imaging Digital Analysis; ODIA = Orthopedic Digital Image Analysis; ICC = 2-way mixed intraclass correlation coefficient; CI = confidence interval; *n* = number; JSW = joint space width; JLCA = joint line convergence angle; KL = Kellgren-Lawrence grade.

**Figure 6.**
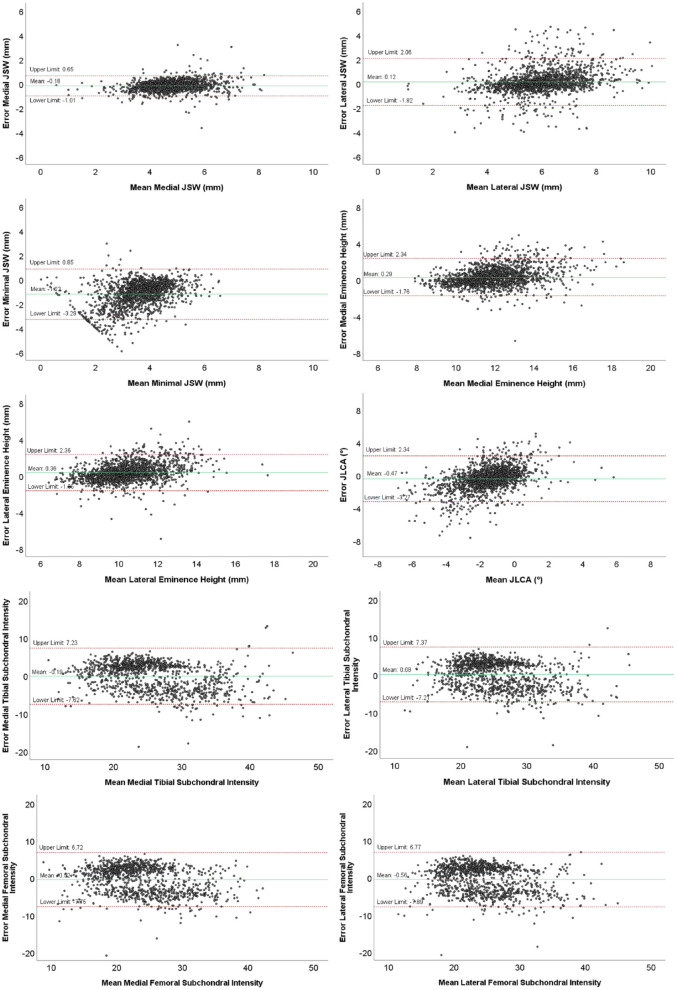
Bland-Altman plots of the measurements performed with ODIA and KIDA, with the means on the horizontal axis and the differences between the 2 measurement methods on the vertical axis. The red lines indicate the 95% limits of agreement, the green line indicates the mean difference. ODIA = Orthopedic Digital Image Analysis; KIDA = Knee Imaging Digital Analysis; JSW = joint space width; JLCA = joint line convergence angle.

Bland-Altman analyses of the JSWs are illustrated in **Figure 6**. The mean difference between the measured mean lateral JSW using ODIA and KIDA was 0.12 mm. The mean difference in the measured mean medial JSW was −0.18 mm, with narrower limits of agreement when compared with the lateral compartment. The mean error of the measured minimal JSW was −1.22 mm, with a systemic error for the smaller minimal JSWs (patients presenting a small minimal JSW). Mean lateral JSW and minimal JSW measurements resulted in higher discrepancies compared with mean medial JSW measurements (**Table 1**).

The Bland-Altman analyses of the eminence height and JLCA measurements using KIDA and ODIA are illustrated in **Figure 6**. The mean difference of the measured JLCA between the 2 methods was low with 0.53° and clearly within the limits of agreement between −3.27° and 2.34°. The mean difference between the measured lateral eminence height was 0.36 mm, and between the measured medial eminence height 0.29 mm. Eminence height measurements of the lateral and medial compartment perform comparably, where higher eminence heights seem to result in bigger differences.

**Figure 6** illustrates the Bland-Altman analyses of the subchondral bone intensity measurements performed using KIDA and ODIA. The mean differences of the subchondral bone intensity measurements were −0.16 mmAl for the lateral tibia and −0.49 mmAl for the medial tibia. The mean differences in measured femoral subchondral bone intensities were −0.51 mmAl for the lateral femur and −0.31 mmAl for the medial femur.

### ODIA Semi-Auto and ODIA Full Auto

ODIA semi-auto and ODIA full auto measurements were compared, yielding results with small deviations. Intraclass correlations and mean absolute errors between the manually checked and full-automated workflow are summarized in **Table 4**.

**Table 4.** ICCs and Mean Absolute Errors With SDs Between ODIA Semi-Auto and ODIA Full Auto.

	ODIA Full Auto 1		
		Mean (SD)	ICC
	Mean lateral JSW	0.43 mm (SD 0.56 mm)	0.805
	Mean medial JSW	0.20 mm (SD 0.21 mm)	0.962
	Minimal JSW	0.27 mm (SD 0.35 mm)	0.905
	JLCA	0.40° (SD 0.56°)	0.904
ODIA semi-auto	Lateral eminence height	0.64 mm (SD 0.73 mm)	0.711
(*n* = 145)	Medial eminence height	0.41 mm (SD 0.51 mm)	0.901
	Medial tibial intensity	0.10 mmAl (SD 0.16 mmAl)	0.999
	Lateral tibial intensity	0.17 mmAl (SD 0.24 mmAl)	0.998
	Medial femoral intensity	0.07 mmAl (SD 0.08 mmAl)	1.000
	Lateral femoral intensity	0.09 mmAl (SD 0.09 mmAl)	1.000

ICC = intraclass correlation coefficient; SD = standard deviation; ODIA = Orthopedic Digital Image Analysis; JSW = joint space width; JLCA = joint line convergence angle.

Reproducibility analyses of the ODIA full auto measurements are listed in **Table 5**. All measurements showed perfect reliability and agreement.

**Table 5.** ICCs and Mean Absolute Errors With SDs Between the 2 ODIA Full Auto Measurements for Reproducibility Analyses.

	ODIA Full Auto 2		
		Mean error (SD)	ICC
	Mean lateral JSW	0.00 mm (SD 0.00 mm)	1.000
	Mean medial JSW	0.00 mm (SD 0.00 mm)	1.000
	Minimal JSW	0.00 mm (SD 0.00 mm)	1.000
	JLCA	0.00° (SD 0.00°)	1.000
ODIA full auto 1	Lateral eminence height	0.00 mm (SD 0.00 mm)	1.000
(*n* = 145)	Medial eminence height	0.00 mm (SD 0.00 mm)	1.000
	Medial tibial intensity	0.00 mmAl (SD 0.00 mmAl)	1.000
	Lateral tibial intensity	0.00 mmAl (SD 0.00 mmAl)	1.000
	Medial femoral intensity	0.00 mmAl (SD 0.00 mmAl)	1.000
	Lateral femoral intensity	0.00 mmAl (SD 0.00 mmAl)	1.000

ICC = intraclass correlation coefficient; SD = standard deviation; ODIA = Orthopedic Digital Image Analysis; JSW = joint space width; JLCA = joint line convergence angle.

## Discussion

This study showed that ODIA can perform quantitative measurements automatically and quickly on knee radiographs. One of the advantages of ODIA is that it is very consistent in its output generation as the method has minimal dependency on the reader. Only the 99-points model derived by the BoneFinder software requires some user interaction.^12,13^ However, this interaction is mostly concerned with verification as the BoneFinder is “trained” with radiographic knee images and—in our case—works mostly automated.^12,13^ Occasionally points need small adjustment to pinpoint them exactly on the bony edge.

This study also tested a full-automated workflow, the ODIA full auto method. This method only required a setup time of around 10 minutes independent of cohort size. To put this into perspective, a well-known manual method was reported to take up to 10 minutes per radiograph.^3^ The results of ODIA full auto and ODIA semi-auto were very similar. This was expected given the very accurate automated segmentations proven by Lindner *et al.* when using BoneFinder®.^12,13^ So, the manual checking of the segmentation did not improve to the accuracy of ODIA, and in other words, the ODIA full auto method accuracy is sufficient for large cohort studies. Also, the reproducibility of the ODIA full auto method was superior to known manual methods, as the absolute differences between the 2 measurement methods was zero.^3^

JSW is often used for monitoring OA in terms of structural tissue progression.^3,6^ In particular, minimal JSW has been used in many clinical OA follow-up studies where a sensitivity of around 0.2 mm has been reported to detect a yearly joint space narrowing.^3,6^ Our ODIA method presented moderate/poor correlations with the KIDA method (lateral and minimal JSW), where there exists a systematic error in the smaller minimal JSW measurements. Underlying reason for discrepancy in minimal/lateral JSW measurements is likely caused by the selection of the anterior border of the lateral tibial plateau,^3^ which is hard to distinguish as the posterior and anterior borders overlap due to its convex shape in the AP direction,^21^ while the medial borders are more visible in AP direction due to its more concave shape.^21^ The anterior lateral border of the tibial plateau is marked by BoneFinder® with points 20 to 26.^12,13^ The visible border of the anterior medial tibial plateau is marked with points 9 to 13, and of the posterior border of the medial plateau with points 9, 13, 35, and 36. ODIA is programmed such that it exclusively takes the anterior border of the lateral tibia plateau, a decision that in fact is made during the generation of the 99-point model in BoneFinder.^12,13^

**Figure 7.**
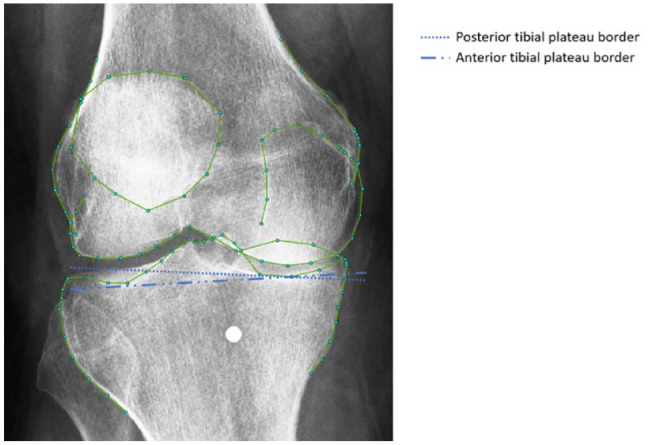
Skewed view of the tibial plateau on a knee radiograph obtained using the Buckland-Wright protocol. There are visible posterior and anterior lateral tibial borders, which are hard to distinguish. Selecting different borders affects the tibial joint line significantly.

The knee radiographs were made using the Buckland-Wright protocol, with the knee in a semi-flexed position (7°-10°) and weightbearing PA view,^16^ compensating for the average tibial slope and ensuring a parallel view of the tibial plateau.^17^ However, it is most likely that not all patients fall within the standard deviation of the reported tibial slope average and were not exactly placed in 7°-10° of knee flexion, leading to a skewed view of the tibial plateau with visible posterior and anterior borders. In **Figure 7**, this can be observed, where BoneFinder® annotated the correct anterior instead of the also visible incorrect posterior border.^12,13^ Therefore, the mean lateral JSW measurements performs worse compared with mean medial JSW measurements in terms of reported ICCs between KIDA and ODIA. ICCs of the measured mean medial JSW (KIDA and ODIA) are all good to excellent as there is no distrust on the anterior bone edge. The cases with the largest differences in lateral/minimal JSW measurements between ODIA and KIDA highlighted that the choice of the anterior tibial joint line was indeed the problem.

Erroneous placement of the anterior tibial border would also affect the JLCA measurement; hence, this line forms the basis for the measurement at the tibial side of the joint.^17^ ODIA and KIDA measurements of the JLCA are different when the lateral anterior joint border cannot be selected clearly, like displayed in **Figure 7**. In addition to the discrepancy in JLCA ratings caused by the selection of the anterior lateral tibial border, the used landmarks for the JLCA measurements could also influence the moderate ICC. Marijnissen *et al.* reported the JLCA measurements as the joint angle.^3^ They used the line which is tangent to the most convex points on the femoral condyles, and a line touching the most distal concaves of the tibial plateau subchondral line.^3^ This method corresponds with the reported lower limb geometry measurement principles of Paley.^17^ Another method for the tibial joint line, very often used by clinicians, is a line running tangent to the most proximal tibial articular surfaces.^8,22^-^24^ ODIA achieved this by selecting landmark 7 and 26 of the BoneFinder® segmentation, and it draws a line between those points (**Fig. 2**).^12,13^

The measurements of subchondral bone intensities of the 2 femoral and tibial compartments reported good ICCs between the KIDA and ODIA measurements. Subchondral bone intensity measurements could be very important in early OA detection methods, as early stage OA is associated with changes in bone mineralization and bone volume, via increased bone turnover.^25^ This will change the subchondral intensities on radiographs.^2,9^ A review summarized the role of subchondral bone in the development of OA, changes in these structures were even observed before the presence of cartilage legions. Moreover, there is a strong evidence that there is an association between OA and subchondral bone mineral density.^26^ Therefore, intensity measurements are potentially valuable in early OA detection as these subchondral bone changes occur even before the onset of cartilage degeneration.^26^ There was a small difference between the reported intensities in KIDA and ODIA likely because of small differences in choice of location of the subchondral regions.

An important limitation of the current study is that there is no method to validate the ODIA measurements with 3D determined parameters, as 2D measurements are always prone to knee rotation and flexion bias. However, the annotated landmark points used for the automated ODIA measurements were meticulously checked by an experienced observer, making sure that the radiographs were segmented correctly.

Finally, it is good to realize that 2D JSW measurement will always have problems with validity, as the JSW is measured between 2 lines that are not a direct true opposite. Therefore, the 2D radiographic definition of JSW—in particular, minimum JSW—is an artificial number. A suggestion to solve JSW measurement inaccuracies caused by the selection of tibial plateau borders is implementing 3D technology, as this problem is inherent with 2D imaging. A weightbearing computed tomography (CT) scan of the knee joint would be valuable in both research and clinical applications.^27^ Only this way, the true JSW can be measured and defined over time. Techniques with CT image registration onto weightbearing knee radiographs are therefore of great value.

Besides the current general OA radiographic markers, ODIA enables to easily generate other parameters that are directly or indirectly related to OA. For example, varus and valgus can be determined using FTA measurements.^7^ Likewise, bone geometry (mechanical medial proximal tibial angle and mechanical lateral distal femoral angle) and its relation to OA progression and incidence are valuable in research.^28^ The method of this current study can be expanded with additional parameters in future projects. For instance, knee geometry measurements can be further expanded with the measurement of the medial proximal tibial angle and the lateral distal femoral angle.

## Conclusion

The current ODIA tool provides important measurements of OA parameters in an automated manner from standard radiographs of the knee. Given the automated and computerized methodology that has very high reproducibility, ODIA is suitable for large epidemiological cohorts with various follow-up time points to investigate structural progression, such as CHECK or OAI.^15^
